# Multigene Hereditary Cancer Panels Reveal High-Risk Pancreatic Cancer Susceptibility Genes

**DOI:** 10.1200/PO.17.00291

**Published:** 2018-07-25

**Authors:** Chunling Hu, Holly LaDuca, Hermela Shimelis, Eric C. Polley, Jenna Lilyquist, Steven N. Hart, Jie Na, Abigail Thomas, Kun Y. Lee, Brigette Tippin Davis, Mary Helen Black, Tina Pesaran, David E. Goldgar, Jill S. Dolinsky, Fergus J. Couch

**Affiliations:** **Chunling Hu**, **Hermela Shimelis**, **Eric C. Polley**, **Jenna Lilyquist**, **Steven N. Hart**, **Jie Na**, **Abigail Thomas**, **Kun Y. Lee**, and **Fergus J. Couch**, Mayo Clinic, Rochester, MN; **Holly LaDuca**, **Brigette Tippin Davis**, **Mary Helen Black**, **Tina Pesaran**, and **Jill S. Dolinsky**, Ambry Genetics, Aliso Viejo, CA; and **David E. Goldgar**, University of Utah, Salt Lake City, UT.

## Abstract

**Purpose:**

The relevance of inherited pathogenic mutations in cancer predisposition genes in pancreatic cancer is not well understood. We aimed to assess the characteristics of patients with pancreatic cancer referred for hereditary cancer genetic testing and to estimate the risk of pancreatic cancer associated with mutations in panel-based cancer predisposition genes in this high-risk population.

**Methods:**

Patients with pancreatic cancer (N = 1,652) were identified from a 140,000-patient cohort undergoing multigene panel testing of predisposition genes between March 2012 and June 2016. Gene-level mutation frequencies relative to Exome Aggregation Consortium and Genome Aggregation Database reference controls were assessed.

**Results:**

The frequency of germline cancer predisposition gene mutations among patients with pancreatic cancer was 20.73%. Mutations in *ATM*, *BRCA2*, *CDKN2A*, *MSH2*, *MSH6*, *PALB2*, and *TP53* were associated with high pancreatic cancer risk (odds ratio, > 5), and mutations in *BRCA1* were associated with moderate risk (odds ratio, > 2). In a logistic regression model adjusted for age at diagnosis and family history of cancer, *ATM* and *BRCA2* mutations were associated with personal history of breast or pancreatic cancer, whereas *PALB2* mutations were associated with family history of breast or pancreatic cancer.

**Conclusion:**

These findings provide insight into the spectrum of mutations expected in patients with pancreatic cancer referred for cancer predisposition testing. Mutations in eight genes confer high or moderate risk of pancreatic cancer and may prove useful for risk assessment for pancreatic and other cancers. Family and personal histories of breast cancer are strong predictors of germline mutations.

## INTRODUCTION

Pancreatic cancer (PC) is the fourth most common cause of death resulting from cancer in the United States.^1^ Epidemiologic studies have suggested that 10% to 20% of PCs are associated with an inherited component, with familial PC, defined as kindreds containing at least two affected first-degree relatives, as an established entity of inherited disease.^2^ PC is a component of hereditary breast-ovarian cancer syndrome,^3,4^ Lynch syndrome,^5,6^ familial adenomatous polyposis,^7^ familial atypical multiple mole melanoma syndrome,^8^ hereditary pancreatitis,^9^ Peutz-Jeghers syndrome,^10^ and Li-Fraumeni syndrome.^11^ Recent studies involving familial PC kindreds have further characterized the role of *BRCA1*/*2*, *CDKN2A*, *ATM*, and *PALB2* in PC susceptibility.^12-14^ Until recently, germline studies of PCs have focused on single cancer predisposition genes.^15,16^ The first panel-based study of 13 cancer predisposition genes among patients with PC identified 11 mutations (3.8%) in *ATM*, *BRCA1*/*2*, *MLH1*, *MSH2*, *MSH6*, and *TP53*,^17^ whereas a 22-gene panel–based study identified *ATM*, *BRCA1*/*2*, *CHEK2*, and *PALB2* mutations in 13% of 96 sequentially collected PCs.^18^ A majority of these mutations were identified in PCs with a family history of pancreatic, breast, ovarian, or colorectal cancer, suggesting that a better understanding of PC risk will depend on evaluation of families with broad constellations of tumors.^18^ More recently, panel-based approaches identified germline mutations in 4% (33 of 854) of patients with apparently sporadic PC^19^ and in 25% (44 of 176) of patients with advanced PC.^20^ Here, we report results from panel-based clinical testing of 1,652 patients with PC from a large cohort of > 140,000 patients evaluated by a single diagnostic laboratory and calculate gene-specific risks of PC by comparison with Exome Aggregation Consortium (ExAC) and Genome Aggregation Database (gnomAD) reference controls.^21,22^

## METHODS

### Study Population

Patients with PC (N = 1,819) were identified from a large cohort of > 140,000 patients undergoing multigene panel testing of seven to 49 cancer predisposition genes between March 2012 and June 2016 at Ambry Genetics^23^ (Aliso Viejo, CA; Appendix Table A1). Demographic and personal and family cancer history information was provided by the ordering clinician using test requisition forms, clinic notes, and pedigrees. Clinical histories and molecular results were reviewed and summarized. Exclusion criteria, including the presence of neuroendocrine tumors or intraductal papillary mucinous neoplasms, reduced the number of patients for analysis (N = 1,652; Appendix). The study was approved by the Western Institutional Review Board.

### Multigene Panel Testing

Mutation testing was performed by sequencing of targeted custom capture products from several multigene panels and targeted chromosomal microarray analysis, as previously described.^24^ Genomic DNA was isolated from each patient’s blood or saliva specimen using a standardized methodology (Qiagen, Valencia, CA). Sequence enrichment was performed by incorporating the genomic DNA into microfluidics chip or microdroplets along with primer pairs or by a bait-capture methodology using long biotinylated oligonucleotide probes (RainDance Technologies, Billerica, MA; Integrated DNA Technologies, San Diego, CA), followed by polymerase chain reaction and then next-generation sequencing analysis (Illumina, San Diego, CA) of all coding exons plus at least five bases into the 5′ and 3′ ends of all the introns and untranslated regions. A targeted chromosomal microarray was used for the detection of gross deletions and duplications for all genes except *PMS2* (Agilent, Santa Clara, CA). Gross deletion and duplication analysis of *PMS2* was performed using MLPA kit #P008-B1 (MRC-Holland, Amsterdam, the Netherlands) and Sanger sequencing. Initial data processing and base calling were performed using RTA 1.12.4 (HiSeq Control software [version 1.4.5]; Illumina). Sequence quality filtering at Q20 was executed with CASAVA software (version 1.8.2; Illumina, Hayward, CA). Sequence fragments were aligned to the reference human genome (GRCh37), and variant calls were generated using CASAVA. Mutations were annotated with the Ambry Variant Analyzer, a proprietary alignment and variant annotation software (Ambry Genetics). All mutations identified by Ambry Genetics are submitted to the ClinVar public database.

### Statistical Methods

The observed frequency of all pathogenic mutations within each gene in white patients with PC was compared with the frequency of pathogenic mutations in the ExAC non-Finnish European (NFE) non–The Cancer Genome Atlas (TCGA) reference control after data cleaning and filtering (Appendix) as previously described.^23^ Copy number variants in all genes and mutations in pseudogene homology regions (*PMS2* exons 9 and 11 to 15) were excluded from cases and controls for risk estimation, because these alterations were not individually validated in ExAC or gnomAD controls. Established low-penetrance mutations (eg, *APC* p.Ile1307Lys) were excluded. Associations between combined mutations in each gene and PC were estimated by odds ratios (ORs) and corresponding 95% CIs based on Fisher’s exact test. *P* values < .05 were considered statistically significant. Genes were categorized as high risk (OR, > 5.0), moderate risk (OR, 2.0 to 5.0), or of no clinical relevance (OR, < 2.0). Similar studies were conducted using a combined gnomAD NFE and gnomAD Ashkenazi Jewish reference control data set, henceforth referred to as gnomAD. Although these gnomAD controls partially overlap with ExAC NFE non-TCGA controls, the substantially increased number along with updated variant calling algorithms identified gnomAD as an independent reference control data set. Sensitivity analyses for associations were performed for associations between genes and age at diagnosis; cases of PC tested with a targeted PC panel; all races and ethnicities combined; personal history of breast cancer or melanoma; family history of PC, breast cancer, ovarian cancer, uterine or endometrial cancer, melanoma, or colorectal cancer; and mutations meeting strict PASS criteria in ExAC.^25^ Associations between mutations and age at PC diagnosis were evaluated using the Kolmogorov-Smirnov test. Associations with personal and family histories of other cancers were also evaluated by logistic regression, with adjustment for family history and age at diagnosis.

## RESULTS

### Characteristics of Study Population

The phenotypic characteristics of 1,652 patients with PC of all races and ethnicities and those of 1,256 white patients are listed in Table 1. Compared with a median age at PC diagnosis of 70 years in Surveillance, Epidemiology, and End Results registries between 2010 and 2014,^26^ the median age at diagnosis was 63 years among patients with PC. PC was the first or only cancer diagnosed in 915 (72.9%) white patients with PC. Pathology was reported for 16.9% of patients, with the majority reported as adenocarcinoma (95.7%). Among white patients with PC, 38.1% had a first- or second-degree relative with PC, and 48.8% had a family history of breast cancer (Table 1). Similar frequencies were observed for patients with PC of all races and ethnicities.

**Table 1.**
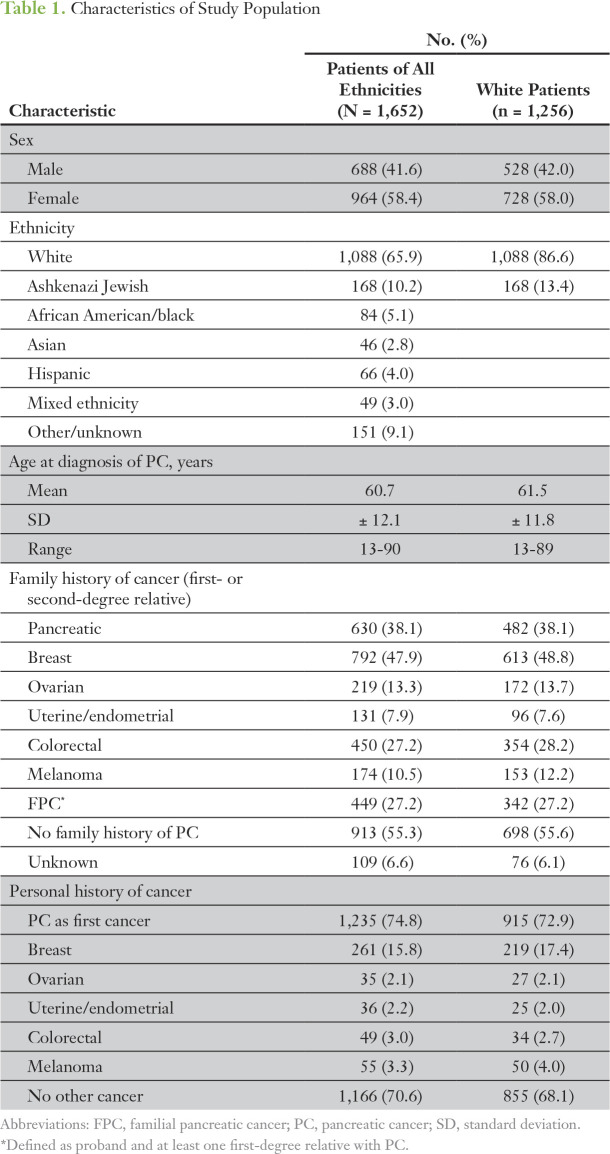
Characteristics of Study Population

### Pathogenic Mutations Among Patients With PC

The combined frequency of mutations in genes from all hereditary cancer testing panels was 20.73% for patients with PC of any race or ethnicity and 21.12% for white patients (Appendix Table A2). *ATM* (3.79%), *BRCA2* (3.72%), *CHEK2* (2.31%), *PALB2* (1.89%), and *CDKN2A* (1.32%) had the highest frequencies of pathogenic mutations among white patients with PC (Appendix Table A2). In contrast, mutations in mismatch repair genes were relatively rare (*MSH6* [1.01%], *MSH2* [0.25%], *MLH1* [0.08%], and *PMS2* [0.08%]). Eight patients had more than one mutation (Appendix Table A3), including a *CDKN2A* c.71G>C (p.Arg24Pro) homozygote. *BRCA2* was the most frequently mutated predisposition gene (4.64%) among patients diagnosed at age ≤ 63 years, and *ATM* was most frequently mutated (4.03%) in patients with PC diagnosed at age > 63 years (Appendix Table A4). Only mutations in *BRCA2* (median age at diagnosis, 56 years) were associated with a younger age at diagnosis compared with all patients with PC (*P* = .001).

### Associations Between Pathogenic Mutations and PC

Mutations in *ATM*, *BRCA2*, *CDKN2A*, *MSH2*, *MSH6*, *PALB2*, and *TP53* were significantly associated with high risk of PC (OR, > 5), whereas deleterious mutations in *CHEK2* and *BRCA1* were associated with moderate risk (OR, > 2; Table 2). Results for all panel genes are listed in Appendix Table A5. Association analyses using gnomAD reference controls confirmed all significant associations, and gene-specific risk estimates were highly similar, except for slightly attenuated risk for *PALB2* mutations and increased risk for *TP53* (Appendix Table A6).

**Table 2.**
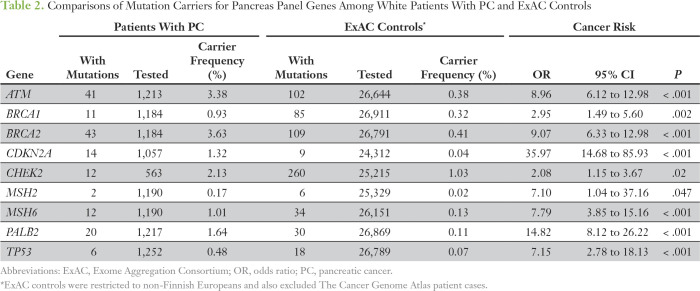
Comparisons of Mutation Carriers for Pancreas Panel Genes Among White Patients With PC and ExAC Controls

The same genes were associated with increased PC risk when considering patients of all races and ethnicities compared with ExAC all race and ethnicity controls (Appendix Table A7) and after excluding those who had previously tested negative for *BRCA1*/*2* mutations before panel testing (Appendix Table A8). Risk estimates for most genes were slightly diminished when including only those patients with PC for whom PC was the first cancer diagnosis, although *MSH2* and *TP53* mutations were no longer significantly associated with moderate risk of PC because of the decreased number of mutations in patients with PC, and the modest OR associated with *CHEK2* was marginally significant (Appendix Table A9). In contrast, analyses using only ExAC NFE non-TCGA variants in the high-quality PASS category marginally increased the ORs for each gene (Appendix Table A10). Sensitivity analyses were also performed after excluding patients with PC with a family history of breast, ovarian, endometrial, colorectal, melanoma, or pancreatic cancer (Appendix Tables A11 to A16, respectively).

### Characteristics of PCs With Mutations in PC Predisposition Genes

The frequency of mutations in the high- and moderate-risk PC predisposition genes was increased in patients with PC with a personal history of breast cancer (Table 3), with almost two-fold more mutations observed in *ATM* (6.80%), *BRCA2* (6.50%), *PALB2* (3.38%), *BRCA1* (2.00%), and *TP53* (0.91%). Results from logistic regression analysis confirmed these findings for *ATM* (*P* = .0065) and *BRCA2* (*P* = .0092; Table 4). In contrast, mutations in the mismatch repair genes *CHEK2* and *CDKN2A* collectively decreased from 4.89% to 2.52% in the context of personal history of breast cancer (Table 3). Mutations in *ATM*, *BRCA2*, and *PALB2* were also more frequent in patients with PC with a family history of breast cancer (first- or second-degree relative; Table 3). In contrast, only *PALB2* and *MSH2* displayed a substantial increase in mutation frequency among patients with a family history of PC, and only *CHEK2*, *MSH2*, and *TP53* had increased frequencies of mutation among patients with PC with a family history of colorectal cancer (Table 3). Results from logistic regression analysis confirmed the association of *PALB2* mutations with family history of PC (*P* = .029) or breast cancer (*P* = .0056) and the association of *CHEK2* mutations with family history of colorectal cancer (*P* = .014; Table 4).

**Table 3.**
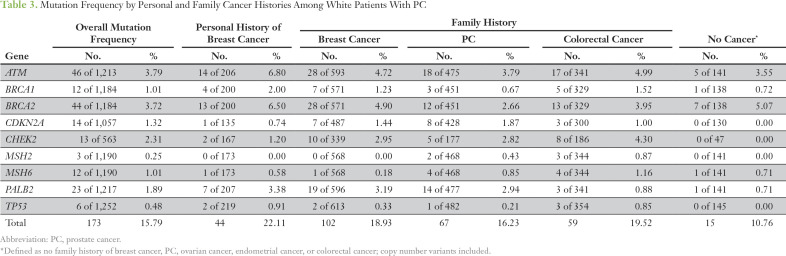
Mutation Frequency by Personal and Family Cancer Histories Among White Patients With PC

**Table 4.**
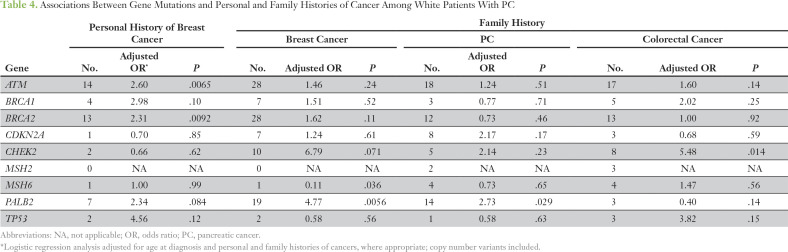
Associations Between Gene Mutations and Personal and Family Histories of Cancer Among White Patients With PC

### Performance of Genetic Testing Criteria Among Mutation Carriers

Consensus clinical genetic testing guidelines include PC as a component tumor for seven of the confirmed PC genes in this study (*BRCA1*/*2*, *MSH2*, *MSH6*, *ATM*, *PALB2*, and *CDKN2A*).^27-29^ Clinical histories of patients with mutations in these genes were evaluated to determine whether the respective genetic testing criteria were met (Table 5). Although a majority of *BRCA1*/*2* and all *MSH2* mutation carriers displayed histories consistent with testing criteria, ≤ 50.0% of *ATM*, *CDKN2A*, *PALB2*, and *MSH6* carriers met criteria. In addition, no *CDKN2A* families met diagnostic criteria for familial atypical multiple mole melanoma syndrome,^30^ and 38.9% (seven of 18) did not report any personal or family history of melanoma.

**Table 5.**
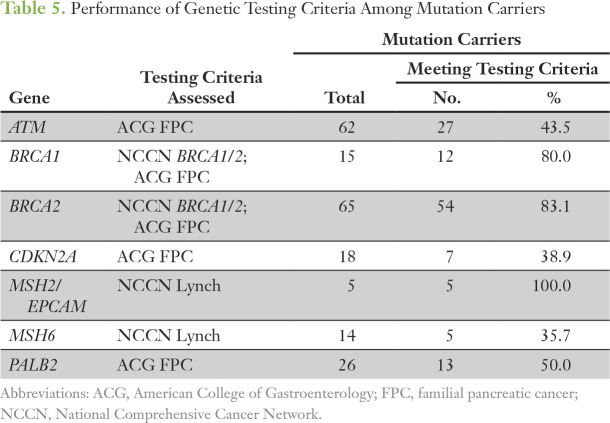
Performance of Genetic Testing Criteria Among Mutation Carriers

## DISCUSSION

Here we report a study of cancer predisposition gene mutations among patients with PC on the basis of a cohort of individuals undergoing hereditary cancer multigene panel testing from a single clinical laboratory. Results from case-control studies of the PC cases and ExAC reference controls identified six genes associated with high risk (OR, > 5) of PC (*ATM*, *BRCA2*, *CDKN2A*, *MSH6*, *PALB2*, and *TP53*), consistent with previous smaller studies and segregation studies from PC families. *MSH2* was also associated with a high risk of PC; however, additional studies are needed to confirm these findings, because this association was based on a limited number of mutations detected among PC cases. There has been some debate regarding the contribution of *BRCA1* mutations to PC risk, because early studies were enriched for founder mutations from Ashkenazi Jewish patients with PC. Here we show that *BRCA1* mutations are associated with a moderate risk (OR, > 2) of PC, even in a series of sensitivity analyses accounting for potential modifying effects of other cancers. *CHEK2* mutations were also associated with a moderate risk of PC; however, this association was either diminished (OR, < 2) or nonsignificant in several sensitivity analyses. In addition, the association of *CHEK2* with PC was attenuated (OR, 1.64; 95% CI, 1.02 to 2.62; *P* = .046) when including the common p.I157T variant in the analyses, consistent with the lower penetrance of this alteration. Given the instability of the risk estimates, additional studies are needed to establish the influence of *CHEK2* mutations on PC risk. Despite the association of *STK11* with high risk of PC, no mutations were detected in this cohort. One likely explanation is that *STK11* mutations are unlikely to occur in the absence of pathognomonic clinical characteristics associated with Peutz-Jeghers syndrome, and therefore, patients with suspected Peutz-Jeghers syndrome may be referred for single-gene testing more often than multigene testing. Pathogenic mutations in other panel genes were still sufficiently uncommon to allow assessment of associations with risk (eg, *APC*, *MLH1*).

The risk estimates for PC associated with each of these established predisposition genes will help improve clinical PC risk assessment. For some genes, these results offer more precise estimates than previously reported, whereas for others, such as *PALB2* and *ATM*, we are the first to characterize the level of risk, to our knowledge. It should be noted that the interpretation of the risks reported here is specific to patients referred for hereditary cancer genetic testing based on a personal or family history of cancer (at least one diagnosis of PC in the family), and thus, these data may not be applicable to the general population or unselected PC cohorts. Despite the enrichment for cases with personal or family history of cancer, these risks are derived from a broader clinical cancer testing cohort compared with previous studies selected for classic syndromic phenotypes such as FAMMM and therefore demonstrate that PC risk from syndromic genes remains high across a range of clinical histories. Furthermore, this enrichment presented an opportunity to explore predictors of germline mutations.

In total, 13% of patients had mutations in genes significantly associated with increased risk for PC across a range of sensitivity analyses (*ATM*, *BRCA1*, *BRCA2*, *CDKN2A*, *MSH6*, *PALB2*, and *TP53*). Consistent with results from a previous study of 96 sequentially recruited patients from the Mayo Clinic,^18^ 90% (158 of 173) of the mutations in the risk-associated genes in this study were from patients with a family history of pancreatic, breast, ovarian, endometrial, or colorectal cancer. Family history of breast, pancreatic, or colorectal cancer was a significant predictor of positive results, suggesting that histories of these cancers should specifically be considered as genetic testing guidelines evolve for PC. The remaining 9% (15 of 173) of mutations were found in the approximately 65% of patients with PC without a family history of these cancers, suggesting a mutation rate of only 2.1% in white patients with PC without a family history of cancer (15 mutations in 698) in the clinically tested cohort. Additional studies of population-based series of patients with PC are needed to determine whether clinical panel testing should be considered for patients with PC unselected for family history.

In practice, patients with PC may not benefit directly from genetic testing because of the high mortality rate for this cancer. However, knowledge of mutation status for genes such as *BRCA1*/*2* and *PALB2* with respect to clinical trial eligibility for targeted agents such as poly (ADP-ribose) polymerase inhibitors may make genetic testing more appealing. In addition, mutation-positive family members can significantly benefit from knowledge of increased risk for a variety of cancers, including PC, and mutation-negative family members can also adjust their cancer screening protocols accordingly. All genes associated with high and moderate PC risk in this study have National Comprehensive Cancer Network guidelines addressing risk management for cancers beyond PC. In addition, the International Cancer of the Pancreas Screening Consortium and the American College of Gastroenterology ^29,31^ recommend that PC surveillance, including annual endoscopic ultrasound and/or magnetic resonance imaging, be considered for individuals with > 5% lifetime or relative risk for PC. With the exception of *TP53*, all genes demonstrating significant association with increased PC risk in this study are addressed in these recommendations. Results from this study suggest that clinicians should consider PC risk when managing *TP53* mutation carriers, particularly in the presence of a family history of PC. In addition, although *BRCA1* mutation carriers with a first- or second-degree relative with PC are included in the list of patients for whom PC screening should be considered, the moderate PC risk categorization for *BRCA1* in this study suggests this may not be clinically indicated.

ExAC NFE non-TCGA controls were used in this study because of the lack of a large series of matched controls. Although the use of large reference data sets is not ideal, the large sample size allows precise estimation of the frequency of mutations in individuals without cancer and is likely reflective of the general population. In addition, we applied many data cleaning steps and used consistent criteria for selection of mutations in the clinical cohort of patients with PC and the ExAC controls to ensure that the data sets were adequately normalized for case-control association analyses. Another potential limitation of this study is the quality of the clinical history information available for patients with PC. In a recent assessment of the quality of clinical history information for patients undergoing hereditary cancer panel testing, pedigrees and/or clinic notes were provided for 46% of randomly selected patient cases (unpublished data). When compared with pedigrees and clinic notes, a vast majority of proband cancers were reported completely (95%) and accurately (> 99%) on test requisition forms. Completeness and accuracy remained high (97%) for PCs reported on test requisition forms. Among family members, 76% of melanomas and > 80% of breast, ovarian, colorectal, endometrial, and pancreatic cancers were reported with ≥ 98% accuracy on test requisition forms. Therefore, the variant frequencies and PC risk estimates presented in this analysis were derived from a laboratory-based cohort with high-quality clinical cancer history information.

Overall, the findings from this large study of PC predisposition gene mutations shed light on the spectrum of mutations that can be expected for patients with PC referred for cancer predisposition testing and identify *ATM*, *BRCA2*, *CDKN2A*, *MSH6*, *PALB2*, and *TP53* as high-risk PC genes that should be considered routinely as part of any comprehensive PC risk evaluation process.

## Figures and Tables

**Table A1.**
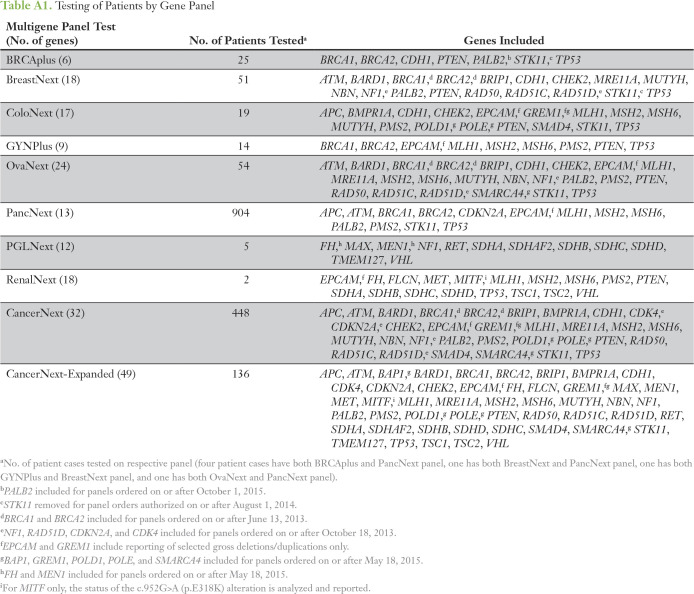
Testing of Patients by Gene Panel

**Table A2.**
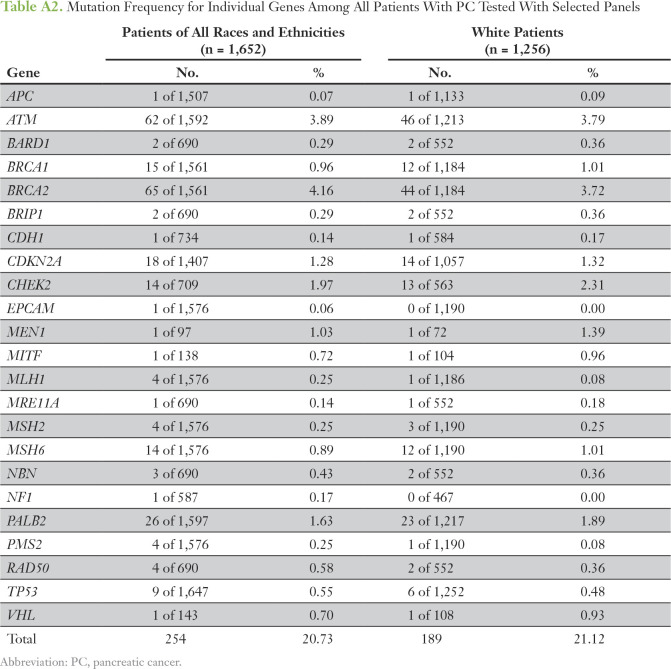
Mutation Frequency for Individual Genes Among All Patients With PC Tested With Selected Panels

**Table A3.**
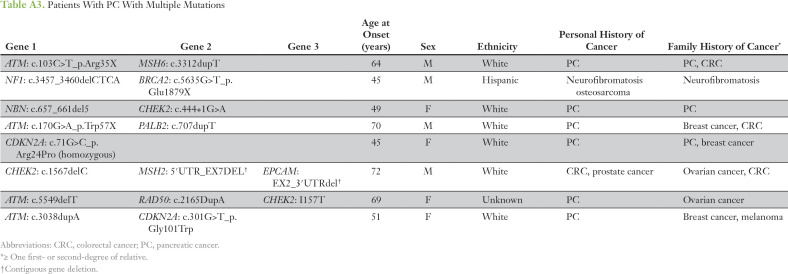
Patients With PC With Multiple Mutations

**Table A4.**
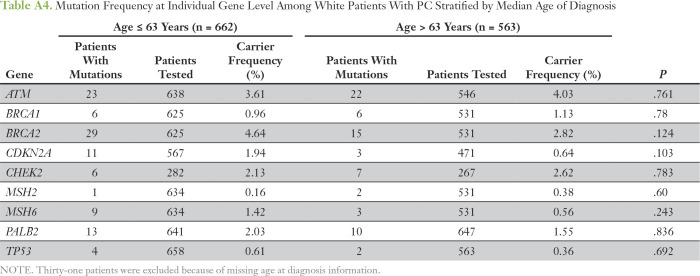
Mutation Frequency at Individual Gene Level Among White Patients With PC Stratified by Median Age of Diagnosis

**Table A5.**
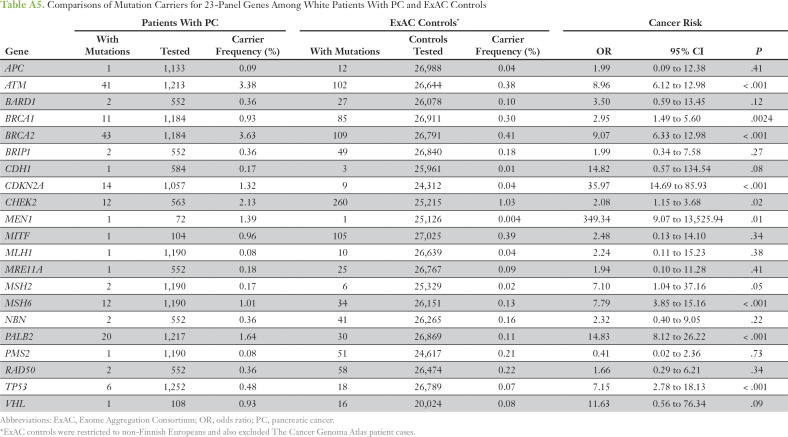
Comparisons of Mutation Carriers for 23-Panel Genes Among White Patients With PC and ExAC Controls

**Table A6.**
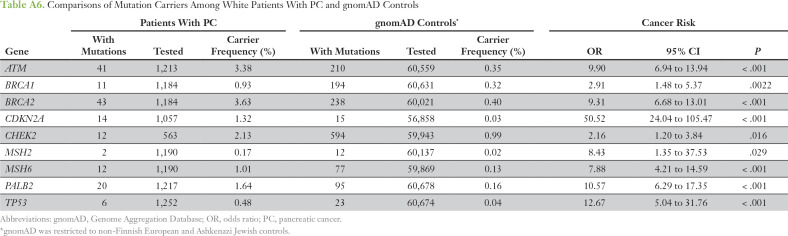
Comparisons of Mutation Carriers Among White Patients With PC and gnomAD Controls

**Table A7.**
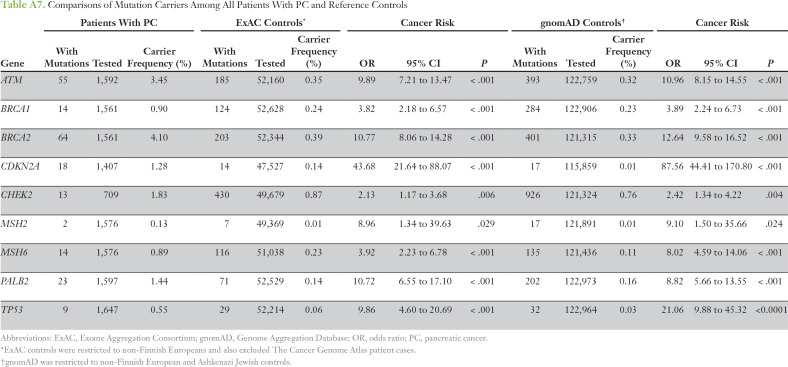
Comparisons of Mutation Carriers Among All Patients With PC and Reference Controls

**Table A8.**
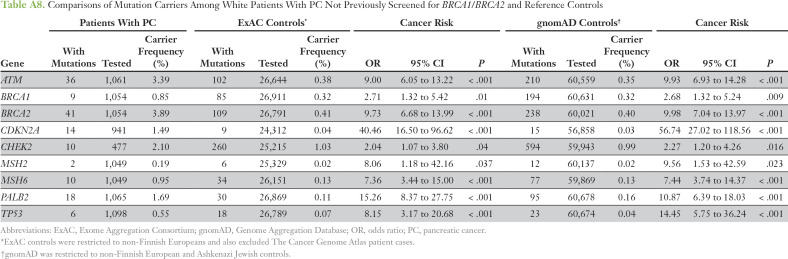
Comparisons of Mutation Carriers Among White Patients With PC Not Previously Screened for *BRCA1*/*BRCA2* and Reference Controls

**Table A9.**
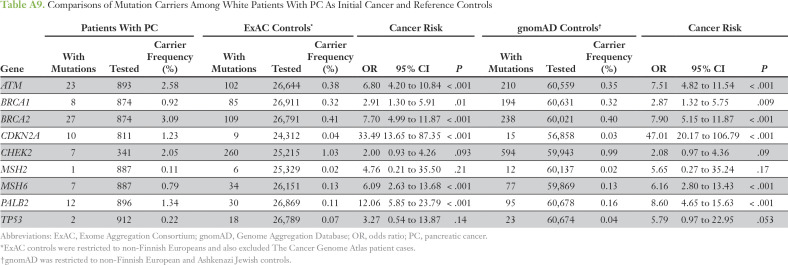
Comparisons of Mutation Carriers Among White Patients With PC As Initial Cancer and Reference Controls

**Table A10.**
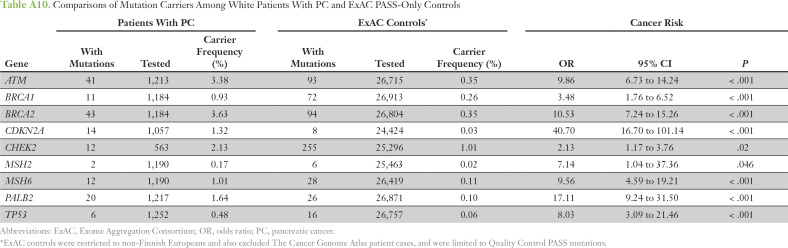
Comparisons of Mutation Carriers Among White Patients With PC and ExAC PASS-Only Controls

**Table A11.**
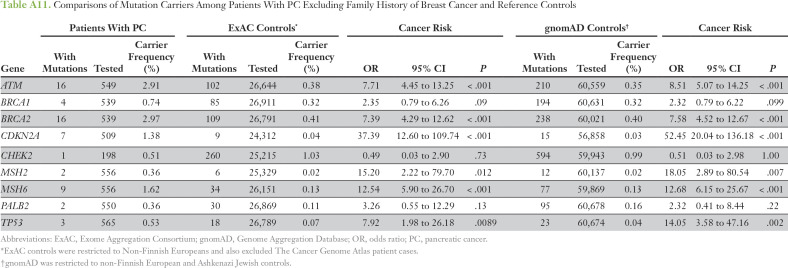
Comparisons of Mutation Carriers Among Patients With PC Excluding Family History of Breast Cancer and Reference Controls

**Table A12.**
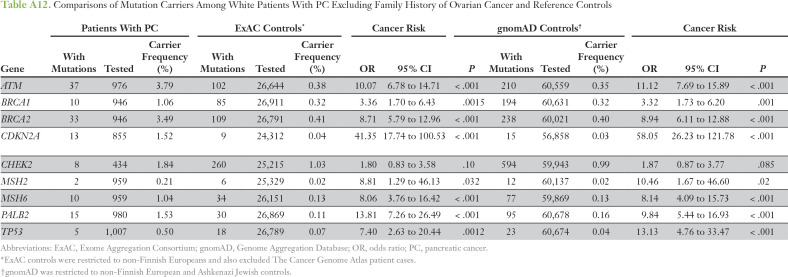
Comparisons of Mutation Carriers Among White Patients With PC Excluding Family History of Ovarian Cancer and Reference Controls

**Table A13.**
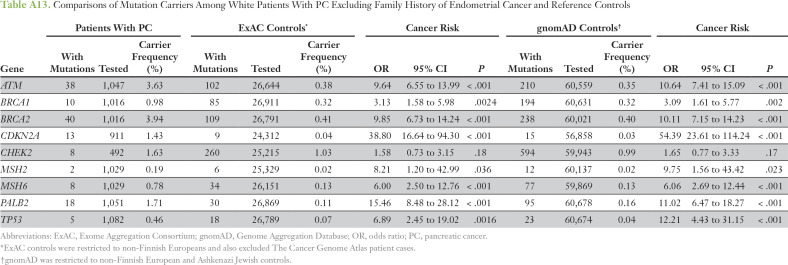
Comparisons of Mutation Carriers Among White Patients With PC Excluding Family History of Endometrial Cancer and Reference Controls

**Table A14.**
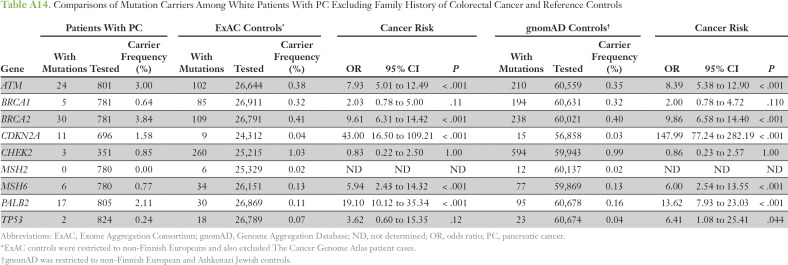
Comparisons of Mutation Carriers Among White Patients With PC Excluding Family History of Colorectal Cancer and Reference Controls

**Table A15.**
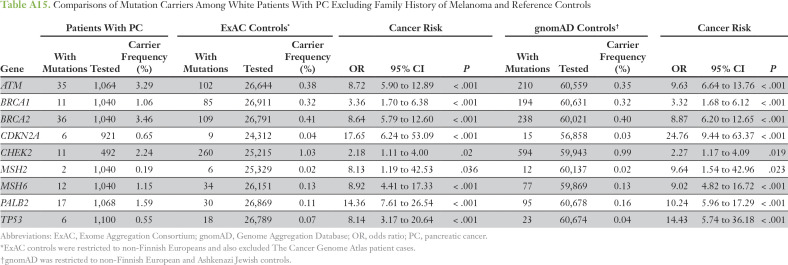
Comparisons of Mutation Carriers Among White Patients With PC Excluding Family History of Melanoma and Reference Controls

**Table A16.**
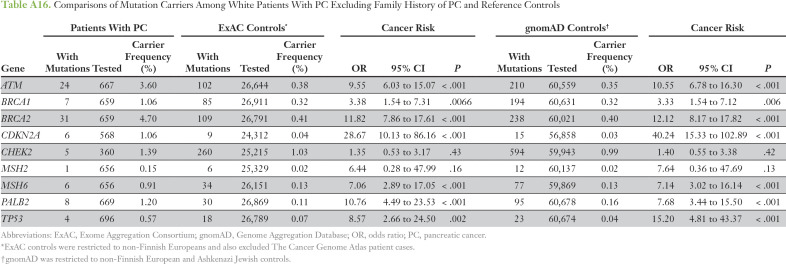
Comparisons of Mutation Carriers Among White Patients With PC Excluding Family History of PC and Reference Controls

## Appendix

### Patient Cases of Pancreatic Cancer

A total of 1,819 patients with pancreatic cancer were identified in a cohort of 140,449 individuals undergoing clinical germline cancer panel testing between March 2012 and June 2016 at a clinical testing laboratory (Ambry Genetics, Aliso Viejo, CA). From these, patients with neuroendocrine or intraductal papillary mucinous neoplasm tumor pathology were excluded, leaving 1,652 patient cases. Mutations derived from testing with all Ambry Genetics panels were used, with PancNext, CancerNext, and CancerNext Expanded panels constituting the majority. All variants classified by Ambry were submitted to ClinVar.

### Exome Aggregation Consortium Reference Controls

The Exome Aggregation Consortium (ExAC) contains exome sequence data from 60,706 unrelated individuals sequenced as part of various disease-specific and population genetic studies. All of the raw data from these projects were reprocessed through a common pipeline. Principal component analysis was performed to identify population clusters corresponding to individuals of European, African, South Asian, East Asian, and admixed American ancestry. Europeans were separated into individuals of Finnish and non-Finnish European (NFE) ancestry. ExAC also contained patient cases of cancer from The Cancer Genome Atlas (TCGA). Exclusion of sequence data from these patient cases yielded ExAC non-NFE non-TCGA reference controls.

### Genome Aggregation Database Reference Controls

The Genome Aggregation Database (gnomAD) contains sequencing data of 123,136 exomes and 15,496 genomes from unrelated individuals sequenced as part of various disease-specific and population genetic studies. The raw sequence data were reprocessed through the same pipeline and jointly variant called to increase consistency across projects. The gnomAD data set contains individuals sequenced using multiple exome capture methods and sequencing chemistries. The resulting variation in coverage was incorporated into the variant frequency calculations for each variant. gnomAD was quality controlled and analyzed using the Hail open-source framework for scalable genetic analysis. gnomAD provides allele frequencies separately for several races and ethnic groups, including non-NFE and Ashkenazi Jewish individuals.

### ExAC Data Cleaning and Filtering

- Restricted to ExAC non-TCGA NFE exome data
- Pathogenic variant classification rules:
  Include all ExAC non-TCGA NFE variants
  Restricted to variants with allele frequency < 0.003, except known pathogenic founder variants (eg, CHEK2 c.1100delC)
  Include loss-of-function variants (nonsense, frameshift, ± 1/2 splice site variants) unless classified as benign or variant of unknown significance by any clinical cancer genetic testing laboratories (Ambry, Sharing Clinical Reports Project, InVitae, GeneDx, Emory, and InSiGHT) in ClinVar.
  Classifications submitted by *Online Mendelian Inheritance in Man*, Breast Cancer Information Core, or other nonclinical groups were not considered in classification criteria. Did not rely on classification in ClinVar submitted before 2010.
  Exclude missense variants and splice site variants beyond ± 1/2 unless classified as pathogenic or likely pathogenic by clinical genetic groups in ClinVar.
  Exclude pathogenic variants with known low risk: APC p.Ile1307Lys, PMS2 c.736_741del6ins11, PTEN p.Pro354Trp, TP53 p.Arg283His, 5′UTR_EX1del, p.Arg181His, p.Arg156His, CHEK2 p.Ile157Thr.
  Exclude pathogenic variants not influenced by nonsense-mediated RNA decay (thresholds: *BRCA2* c.9924, *BARD1* c.1947, *BRIP1* c.2851, *RAD50* c.3698, *RAD51D* c.849).
  Identify variants in *PMS2* pseudogene region (exon 9 and exons 11 to 15); calculate variant frequency and odds ratios without these variants.
  Exclude ExAC non-PASS recurrent variants with allele count in ExAC > eight and tested in < 20,000 ExAC alleles.
  Exclude ExAC non-PASS variants with multiple repetitive sequences called multiple times (eg, *MSH2*_c.942+2_942+6del5, *MSH2_*c.942+2_942+4delTAA, *MSH2_*c.942+2_942+5delTAAA, *MSH2*_c.942+2_942+3delTA, *MSH2*_c.942+2_942+8del7 *MSH2*_c.942+2_942+7del6).
- Allele number was calculated as average of all variants within the coding region of a gene of interest, because different numbers of individuals were tested for each variant.

### gnomAD Data Cleaning and Filtering

- Restricted to gnomAD NFE exome data combined with gnomAD Ashkenazi Jewish exome data.
- Pathogenic variant classification rules:
  Same as in ExAC rules 1 to 8.
  Review variants with allele count ≥ 15 by Integrative Genomics Viewer and by frequency in control data from dbSNP.
- Allele number was calculated as average of all variants within the coding region of a gene of interest. This is important for ExAC, gnomAD, and Ambry patient cases, because different numbers of individuals were tested for each variant.
